# Supplementary material for: Impervious surface and local abiotic conditions influence arthropod communities within urban greenspaces
Source: PeerJ. 2022 Jan 24;10:e12818. doi: 10.7717/peerj.12818 (PMC8793725; doi:10.7717/peerj.12818)
Supplement: Supplemental Information 1 — Arthropods were identified to the 66 morphospecies listed in this table. [file peerj-10-12818-s001.docx]

| **ID** | **Morphospecies Name** | **Order** | **Family** | **Genus** | **Species** | **Count** |
| --- | --- | --- | --- | --- | --- | --- |
| Acar.1 | *Acari Morphospecies 1* | Acari |  |  |  | 4 |
| Aran.1 | *Araneae Morphospecies 1* | Araneae | Dysderidae |  |  | 52 |
| Aran.2 | *Araneae Morphospecies 2* | Araneae | Lycosidae |  |  | 30 |
| Aran.3 | *Araneae Morphospecies 3* | Araneae | Dysderidae |  |  | 45 |
| Aran.4 | *Araneae Morphospecies 4* | Araneae | Pholcidae |  |  | 1 |
| Blat.1 | *Blattodea Morphospecies 1* | Blattodea | Termitoidae | *Reticulitermes* |  | 3 |
| Clad.1 | *Cladocera Morphospecies 1* | Cladocera | Daphniidae | *Daphnia* |  | 1 |
| Col.1 | *Coleoptera Morphospecies 1* | Coleoptera | Cicindelinae | *Cicindela* | *sexguttata* | *2* |
| Col.2 | *Coleoptera Morphospecies 2* | Coleoptera | Curculionidae | *Otiorhynchus* |  | 260 |
| Col.3 | *Coleoptera Morphospecies 3* | Coleoptera | Chrysomelidae | *Cerotoma* |  | 10 |
| Col.4 | *Coleoptera Morphospecies 4* | Coleoptera | Scarabaeidae |  |  | 31 |
| Col.5 | *Coleoptera Morphospecies 5* | Coleoptera | Staphylinidae | *Philonthus* |  | 13 |
| Col.6 | *Coleoptera Morphospecies 6* | Coleoptera | Scarabaeidae | *Popillia* | *japonica* | 1 |
| Col.7 | *Coleoptera Morphospecies 7* | Coleoptera | Carabidae | *Platynus* |  | 7 |
| Col.8 | *Coleoptera Morphospecies 8* | Coleoptera | Cantharidae |  |  | 4 |
| Col.9 | *Coleoptera Morphospecies 9* | Coleoptera | Scarabaeidae |  |  | 2 |
| Dipt.1 | *Diptera Morphospecies 1* | Diptera | Phoridae |  |  | 930 |
| Dipt.2 | *Diptera Morphospecies 2* | Diptera | Muscidae | *Musca* |  | 9 |
| Dipt.3 | *Diptera Morphospecies 3* | Diptera | Syrphidae |  |  | 561 |
| Dipt.4 | *Diptera Morphospecies 4* | Diptera | Dolichopodidae |  |  | 141 |
| Dipt.5 | *Diptera Morphospecies 5* | Diptera | Culicidae | *Aedes* |  | 771 |
| Dipt.6 | *Diptera Morphospecies 6* | Diptera | Drosophilidae | *Drosophila* |  | 163 |
| Dipt.7 | *Diptera Morphospecies 7* | Diptera | Asilidae | *Machimus* |  | 2 |
| Dipt.8 | *Diptera Morphospecies 8* | Diptera | Syrphidae |  |  | 11 |
| Dipt.8 | *Diptera Morphospecies 9* | Diptera | Chironomidae | *Chironomus* |  | 14 |
| Dipt.10 | *Diptera Morphospecies 10* | Diptera | Asilidae | *Laphria* |  | 2 |
| Hem.1 | *Hemiptera Morphospecies 1* | Hemiptera | Membracidae | *Enchenopa* |  | 17 |
| Hem.2 | *Hemiptera Morphospecies 2* | Hemiptera | Cicadellidae |  |  | 673 |
| Hem.3 | *Hemiptera Morphospecies 3* | Hemiptera | Miridae |  |  | 23 |
| Hem.4 | *Hemiptera Morphospecies 4* | Hemiptera | Aphididae | *Aphis* |  | 28 |
| Hem.5 | *Hemiptera Morphospecies 5* | Hemiptera | Aphididae | *Aphis* |  | 66 |
| Hem.6 | *Hemiptera Morphospecies 6* | Hemiptera | Cicadellidae |  |  | 343 |
| Hem.8 | *Hemiptera Morphospecies 7* | Hemiptera | Cicadellidae | *Graphocephala* |  | 1 |
| Hym.1 | *Hymenoptera Morphospecies 1* | Hymenoptera | Formicidae | *Monomorium* |  | 129 |
| Hym.2 | *Hymenoptera Morphospecies 2* | Hymenoptera | Bethylidae |  |  | 110 |
| Hym.3 | *Hymenoptera Morphospecies 3* | Hymenoptera | Vespidae |  |  | 30 |
| Hym.4 | *Hymenoptera Morphospecies 4* | Hymenoptera | Apidae | *Bombus* | *impatiens* | *1* |
| Hym.5 | *Hymenoptera Morphospecies 5* | Hymenoptera | Apidae | *Melissodes* |  | 5 |
| Hym.6 | *Hymenoptera Morphospecies 6* | Hymenoptera | Megachilidae | *Osmia* |  | 16 |
| Hym.7 | *Hymenoptera Morphospecies 7* | Hymenoptera | Halictidae | *Agapostemon* |  | 3 |
| Hym.8 | *Hymenoptera Morphospecies 8* | Hymenoptera | Halictidae | *Sphecodes* |  | 2 |
| Hym.9 | *Hymenoptera Morphospecies 9* | Hymenoptera | Halictidae | *Lasioglossum* |  | 144 |
| Hym.10 | *Hymenoptera Morphospecies 10* | Hymenoptera | Halictidae | *Lasioglossum* |  | 284 |
| Hym.11 | *Hymenoptera Morphospecies 11* | Hymenoptera | Vespidae |  |  | 87 |
| Hym.12 | *Hymenoptera Morphospecies 12* | Hymenoptera | Vespidae | *Polistes* |  | 5 |
| Hym.13 | *Hymenoptera Morphospecies 13* | Hymenoptera | Sphecidae | *Ammophila* |  | 3 |
| Hym.14 | *Hymenoptera Morphospecies 14* | Hymenoptera | Halictidae | *Augochlorella* | *aurata* | *46* |
| Hym.15 | *Hymenoptera Morphospecies 15* | Hymenoptera | Colletidae | *Colletes* | *nudus* | *10* |
| Hym.16 | *Hymenoptera Morphospecies 16* | Hymenoptera | Formicidae | *Camponotus* |  | 32 |
| Hym.17 | *Hymenoptera Morphospecies 17* | Hymenoptera | Apidae | *Apis* | *mellifera* | 9 |
| Hym.18 | *Hymenoptera Morphospecies 18* | Hymenoptera | Megachilidae | *Anthidium* |  | 8 |
| Hym.19 | *Hymenoptera Morphospecies 19* | Hymenoptera | Ichneumonidae |  |  | 19 |
| Hym.20 | *Hymenoptera Morphospecies 20* | Hymenoptera | Colletidae | *Hylaeus* |  | 31 |
| Hym.21 | *Hymenoptera Morphospecies 21* | Hymenoptera | Formicidae | *Camponotus* |  | 14 |
| Hym.22 | *Hymenoptera Morphospecies 22* | Hymenoptera | Halictidae | *Lasioglossum* |  | 19 |
| Hym.23 | *Hymenoptera Morphospecies 23* | Hymenoptera | Colletidae | *Hylaeus* |  | 25 |
| Lep.1 | *Lepidoptera Morphospecies 1* | Lepidoptera | Pieridae | *Pieris* | *rapae* | 2 |
| Lep.2 | *Lepidoptera Morphospecies 2* | Lepidoptera | Nymphalidae | *Phyciodes* |  | 24 |
| Lep.3 | *Lepidoptera Morphospecies 3* | Lepidoptera | Pieridae | *Pontia* |  | 3 |
| Lep.4 | *Lepidoptera Morphospecies 4* | Lepidoptera | Geometridae |  |  | 5 |
| Mant.1 | *Mantodea Morphospecies 1* | Mantodea | Mantidae | *Tenodera* |  | 20 |
| Odon.1 | *Odonata Morphospecies 1* | Odonata | Coenagrionidae | *Enallagma* |  | 6 |
| Orth.1 | *Orthoptera Morphospecies 1* | Orthoptera | Acrididae |  |  | 88 |
| Orth.2 | *Orthoptera Morphospecies 2* | Orthoptera | Gryllidae | *Gryllus* |  | 57 |
| Siph.1 | *Siphonoptera Morphospecies 1* | Siphonoptera |  |  |  | 3 |
| Thys.1 | *Thysanoptera Morphospecies 1* | Thysanoptera | Thripidae | *Thrips* |  | 8 |
