# Supplementary material for: Impervious surface and local abiotic conditions influence arthropod communities within urban greenspaces
Source: PeerJ. 2022 Jan 24;10:e12818. doi: 10.7717/peerj.12818 (PMC8793725; doi:10.7717/peerj.12818)
Supplement: Supplemental Information 2 — When two variables were highly correlated (R ¿±0.7) one of these variables was removed from statistical analysis. We removed “No. Yellow Flowers” and “Site Area” from statistical analyses. Highly correlated variables in the table are in bold. [file peerj-10-12818-s002.docx]

|  | No. Total Flowers | No. Purple Flowers | No. Yellow Flowers | No White Flowers | Temperature | Humidity | Impervious Surface (500 m) | Area-Perimeter Ratio | Site Area |
| --- | --- | --- | --- | --- | --- | --- | --- | --- | --- |
| No. Total Flowers | 1 | 0.563 | **0.840** | 0.653 | -0.294 | 0.081 | -0.071 | 0.147 | 0.289 |
| No. Purple Flowers | 0.563 | 1 | 0.186 | 0.308 | -0.048 | 0.005 | 0.122 | -0.155 | -0.177 |
| No. Yellow Flowers | **0.840** | 0.186 | 1 | 0.258 | -0.291 | -0.038 | 0.018 | 0.070 | 0.276 |
| No. White Flowers | 0.653 | 0.308 | 0.258 | 1 | -0.210 | 0.280 | -0.324 | 0.395 | 0.415 |
| Temperature | -0.294 | -0.048 | -0.291 | -0.210 | 1 | -0.518 | 0.132 | -0.389 | -0.316 |
| Humidity | 0.081 | 0.005 | -0.038 | 0.280 | -0.518 | 1 | -0.002 | 0.126 | -0.102 |
| Impervious Surface (500m) | -0.071 | 0.122 | 0.018 | -0.324 | 0.132 | -0.002 | 1 | -0.662 | **-0.723** |
| Area-Perimeter Ratio | 0.147 | -0.155 | 0.070 | 0.395 | -0.389 | 0.126 | -0.662 | 1 | **0.900** |
| Site Area | 0.289 | -0.177 | 0.276 | 0.415 | -0.316 | -0.102 | **-0.723** | **0.900** | 1 |
