# Supplementary material for: Impervious surface and local abiotic conditions influence arthropod communities within urban greenspaces
Source: PeerJ. 2022 Jan 24;10:e12818. doi: 10.7717/peerj.12818 (PMC8793725; doi:10.7717/peerj.12818)
Supplement: Supplemental Information 3 — Based on this list of candidate models, the top models for each response metric (lowest AICc) were selected. When two environmental factors were within 2 AICc units, the models were considered equivalent and both models were included. [file peerj-10-12818-s003.docx]

| **Response Metric** | **Model** | **AICc** | **R^2^** | **Estimate** | **Standard Error** | **T/Z Value** | **P-Value** |
| --- | --- | --- | --- | --- | --- | --- | --- |
| Abundance | Humidity | 194.04 | 0.39 | 0.036 | 0.01 | 3.543 | <0.001 |
|  | Temperature | 199.05 | 0.17 | -0.024 | 0.012 | -1.901 | 0.057 |
|  | NULL | 199.09 | ---- | 5.834 | 0.079 | 73.97 | ---- |
|  | No. White Flowers | 200.74 | 0.08 | 0.006 | 0.005 | 1.266 | 0.205 |
|  | Impervious Surface | 200.82 | 0.08 | -0.007 | 0.006 | -1.148 | 0.251 |
|  | No. Purple Flowers | 201.1 | 0.06 | -0.006 | 0.006 | -1.04 | 0.299 |
|  | Perimeter-Area Ratio | 202.16 | <0.01 | 0.006 | 0.062 | 0.104 | 0.917 |
|  | No. Total Flowers | 202.16 | <0.01 | < -0.001 | 0.002 | -0.081 | 0.935 |
| Diversity | Humidity | -22.23 | 0.49 | -0.015 | 0.004 | 3.666 | 0.003 |
|  | Temperature | -16.88 | 0.29 | -0.012 | 0.005 | -2.377 | 0.032 |
|  | NULL | -14.54 | --- | 2.518 | 0.034 | 74.03 | --- |
|  | No. Purple Flowers | -13.63 | 0.13 | -0.003 | 0.002 | -1.426 | 0.176 |
|  | No. Total Flowers | -11.69 | 0.01 | -0.0004 | 0.001 | -0.448 | 0.661 |
|  | No. White Flowers | -11.64 | 0.01 | -0.001 | 0.002 | 0.397 | 0.697 |
|  | Impervious Surface | -11.59 | <0.01 | -0.001 | 0.003 | -0.338 | 0.74 |
|  | Perimeter-Area Ratio | -11.55 | <0.01 | 0.008 | 0.028 | 0.282 | 0.782 |
| Richness | No. Purple Flowers | 96.07 | 0.39 | -0.007 | 0.003 | -2.098 | 0.036 |
|  | NULL | 97.95 | --- | 3.424 | 0.045 | 75.87 | --- |
|  | No. Total Flowers | 99.48 | 0.1 | -0.001 | 0.001 | -1.036 | 0.3 |
|  | Perimeter-Area Ratio | 99.63 | 0.08 | 0.035 | 0.035 | 0.98 | 0.327 |
|  | Humidity | 99.8 | 0.07 | 0.007 | 0.008 | 0.879 | 0.379 |
|  | Impervious Surface | 100.25 | 0.03 | -0.002 | 0.004 | -0.575 | 0.565 |
|  | No. White Flowers | 100.38 | 0.02 | 0.001 | 0.003 | 0.455 | 0.649 |
|  | Temperature | 100.49 | <0.01 | -0.002 | 0.008 | -0.341 | 0.753 |
| Hymenoptera Abundance | Temperature | 145.32 | 0.29 | -0.034 | 0.013 | -2.646 | 0.008 |
|  | Perimeter-Area Ratio | 147.25 | 0.2 | 0.117 | 0.06 | 1.95 | 0.051 |
|  | NULL | 147.84 | --- | 4.166 | 0.085 | 49.04 | --- |
|  | Impervious Surface | 148.21 | 0.16 | -0.011 | 0.007 | -1.708 | 0.088 |
|  | Humidity | 148.8 | 0.12 | 0.022 | 0.013 | 1.664 | 0.096 |
|  | No. Total Flowers | 150.71 | 0.01 | -0.001 | 0.002 | -0.463 | 0.643 |
|  | No. Purple Flowers | 150.84 | <0.01 | -0.002 | 0.006 | -0.253 | 0.801 |
|  | No. White Flowers | 150.88 | <0.01 | -0.001 | 0.005 | -0.174 | 0.862 |
| Hemiptera Abundance | Impervious Surface | 163.65 | 0.37 | -0.032 | 0.012 | -2.966 | 0.003 |
|  | Humidity | 165.65 | 0.29 | 0.064 | 0.023 | 2.772 | 0.006 |
|  | No. White Flowers | 167.6 | 0.2 | 0.02 | 0.009 | 2.209 | 0.027 |
|  | NULL | 168.33 | --- | 4.276 | 0.161 | 26.52 | --- |
|  | Perimeter-Area Ratio | 170.13 | 0.07 | 0.131 | 0.123 | 1.07 | 0.285 |
|  | Temperature | 170.9 | 0.03 | -0.019 | 0.028 | -0.693 | 0.488 |
|  | No. Purple Flowers | 171.13 | 0.02 | -0.007 | 0.012 | -0.571 | 0.568 |
|  | No. Total Flowers | 171.38 | <0.01 | 0.001 | 0.004 | 0.158 | 0.874 |
| Diptera Abundance | NULL | 184.93 | --- | 5.092 | 0.11 | 46.3 | --- |
|  | Humidity | 185.75 | 0.13 | 0.03 | 0.017 | 1.749 | 0.08 |
|  | Perimeter-Area Ratio | 186.24 | 0.1 | -0.116 | 0.082 | -1.409 | 0.159 |
|  | Impervious Surface | 187.32 | 0.04 | 0.007 | 0.009 | 0.821 | 0.412 |
|  | Temperature | 186.55 | 0.08 | -0.024 | 0.018 | -1.285 | 0.199 |
|  | No. Purple Flowers | 186.77 | 0.07 | -0.009 | 0.008 | -1.163 | 0.245 |
|  | No. White Flowers | 187.88 | 0.01 | 0.003 | 0.007 | 0.384 | 0.701 |
|  | No. Total Flowers | 188.01 | <0.01 | 0.0001 | 0.003 | -0.052 | 0.958 |
| Hymenoptera Richness | NULL | 75.01 | --- | 2.404 | 0.075 | 31.98 | --- |
|  | Perimeter-Area Ratio | 76.47 | 0.24 | 0.064 | 0.059 | 1.09 | 0.276 |
|  | No. White Flowers | 77.03 | 0.13 | 0.004 | 0.004 | 0.8 | 0.424 |
|  | No. Purple Flowers | 77.3 | 0.07 | -0.003 | 0.006 | -0.586 | 0.558 |
|  | Impervious Surface | 77.42 | 0.05 | -0.003 | 0.006 | -0.473 | 0.636 |
|  | Temperature | 77.54 | 0.02 | -0.004 | 0.013 | -0.331 | 0.741 |
|  | Humidity | 77.61 | <0.01 | 0.003 | 0.012 | 0.203 | 0.839 |
|  | No. Total Flowers | 77.63 | <0.01 | 0.0002 | 0.002 | 0.144 | 0.886 |
| Hemiptera Richness | NULL | 62.75 | --- | 1.447 | 0.121 | 11.93 | --- |
|  | No. Purple Flowers | 63.93 | 0.17 | -0.011 | 0.009 | -1.184 | 0.236 |
|  | No. White Flowers | 64.99 | 0.05 | 0.005 | 0.007 | 0.644 | 0.52 |
|  | No. Total Flowers | 65.01 | 0.04 | -0.002 | 0.003 | -0.597 | 0.55 |
|  | Impervious Surface | 65.1 | 0.03 | 0.005 | 0.01 | 0.537 | 0.591 |
|  | Perimeter-Area Ratio | 65.12 | 0.03 | -0.05 | 0.097 | -0.512 | 0.609 |
|  | Temperature | 65.13 | 0.02 | 0.01 | 0.021 | 0.516 | 0.606 |
|  | Humidity | 65.36 | <0.01 | 0.004 | 0.02 | 0.181 | 0.856 |
| Diptera Richness | NULL | 64.39 | --- | 1.812 | 0.101 | 17.94 | --- |
|  | No. Total Flowers | 65.91 | 0.32 | -0.003 | 0.003 | -1.02 | 0.308 |
|  | No. Purple Flowers | 66.74 | 0.08 | -0.004 | 0.008 | -0.539 | 0.59 |
|  | No. White Flowers | 66.93 | 0.03 | -0.002 | 0.006 | -0.322 | 0.748 |
|  | Perimeter-Area Ratio | 66.93 | 0.03 | -0.025 | 0.08 | -0.314 | 0.754 |
|  | Impervious Surface | 66.95 | 0.02 | 0.002 | 0.008 | 0.286 | 0.775 |
|  | Humidity | 67 | <0.01 | -0.003 | 0.016 | -0.181 | 0.857 |
|  | Temperature | 67.02 | <0.01 | 0.002 | 0.018 | 0.103 | 0.918 |
