# Supplementary material for: Impervious surface and local abiotic conditions influence arthropod communities within urban greenspaces
Source: PeerJ. 2022 Jan 24;10:e12818. doi: 10.7717/peerj.12818 (PMC8793725; doi:10.7717/peerj.12818)
Supplement: Supplemental Information 4 — Based on this list of models, the top models for each response metric (lowest AICc) were selected. We used this list of models to compare whether additive and interactive were better (lower AICc) at explaining the response than solely a univariate model. [file peerj-10-12818-s004.docx]

| **Response Metric** | **Model** | **Estimate** | **Standard Error** | **T/Z Value** | **P**  **Value** | **AICc** |
| --- | --- | --- | --- | --- | --- | --- |
| Abundance | Humidity | 0.036 | 0.01 | 3.543 | <0.001 | 194.04 |
|  | Humidity +  Temperature | 0.031 | 0.013 | 2.673 | 0.008 | 196.86 |
|  |  | -0.011 | 0.012 | -0.890 | 0.374 |  |
|  | Humidity +  Site Type | 0.036 | 0.010 | 3.599 | <0.01 | 197.03 |
|  |  | -0.098 | 0.121 | -0.813 | 0.416 |  |
|  | Humidity × Temperature | 0.003 | 0.002 | 1.782 | 0.075 | 198.44 |
|  | NULL | 5.834 | 0.079 | 73.97 | ---- | 199.09 |
|  | Humidity × Site Type | -0.014 | 0.027 | -0.538 | .591 | 201.12 |
| Diversity | Humidity | -0.015 | 0.004 | 3.666 | 0.003 | -22.23 |
|  | Humidity  + Site Type | 0.015 | 0.004 | 3.570 | 0.586 | -18.97 |
|  |  | 0.029 | 0.052 | 0.558 | 0.586 |  |
|  | Humidity × Site Type | -0.001 | 0.012 | -0.076 | 0.941 | -14.61 |
|  | NULL | 2.518 | 0.034 | 74.03 | --- | -14.54 |
| Richness | No. Purple Flowers | -0.007 | 0.003 | -2.098 | 0.036 | 96.07 |
|  | NULL | 3.424 | 0.045 | 75.87 | --- | 97.95 |
|  | No. Purple Flowers  + Site Type | -0.007 | 0.003 | -2.086 | 0.037 | 99.14 |
|  |  | 0.006 | 0.091 | 0.067 | 0.947 |  |
|  | No. Purple Flowers × Site Type | 0.007 | 0.009 | 0.811 | 0.417 | 102.12 |
| Hymenoptera Abundance | Temperature | -0.034 | 0.013 | -2.646 | 0.008 | 145.32 |
|  | Perimeter-Area Ratio | 0.117 | 0.06 | 1.95 | 0.051 | 147.25 |
|  | Temperature  + Perimeter-Area Ratio | -0.027 | 0.013 | -2.070 | 0.039 | 147.32 |
|  |  | 0.075 | 0.057 | 1.303 | 0.193 |  |
|  | Temperature +  Site Type | -0.028 | 0.013 | -2.175 | 0.030 | 147.39 |
|  |  | 0.187 | 0.145 | 1.291 | 0.197 |  |
|  | NULL | 4.166 | 0.085 | 49.04 | --- | 147.84 |
|  | Perimeter-Area Ratio + Site Type | 0.081 | 0.079 | 1.027 | 0.304 | 150.14 |
|  |  | 0.160 | 0.202 | 0.792 | 0429 |  |
|  | Temperature × Perimeter-Area Ratio | -0.011 | 0.014 | -0.779 | 0.436 | 151.10 |
|  | Temperature × Site Type | -0.010 | 0.028 | -0.349 | 0.728 | 151.65 |
|  | Perimeter-Area Area × Site Type | -0.260 | 0.159 | -1.632 | 0.103 | 151.72 |
| Hemiptera Abundance | Impervious Surface  + Humidity | 0.029 | 0.009 | -3.220 | 0.001 | 161.21 |
|  |  | 0.054 | 0.018 | 3.033 | 0.002 |  |
|  | Impervious Surface | -0.032 | 0.012 | -2.966 | 0.003 | 163.65 |
|  | Impervious Surface × Humidity | -0.003 | 0.002 | -1.420 | 0.156 | 163.59 |
|  | Humidity | 0.064 | 0.023 | 2.772 | 0.006 | 165.65 |
|  | Impervious Surface +  Site Type | -0.371 | 0.011 | -3.311 | 0.001 | 166.14 |
|  |  | -0.299 | 0.265 | -1.128 | 0.259 |  |
|  | NULL | 4.276 | 0.161 | 26.52 | --- | 168.33 |
|  | Humidity +  Site Type | 0.065 | 0.023 | 2.859 | 0.004 | 168.88 |
|  |  | 0.176 | 0.272 | 0.647 | 0.517 |  |
|  | Impervious Surface × Site Type | -0.026 | 0.023 | -1.125 | 0.261 | 169.20 |
|  | Humidity × Site Type | -0.053 | 0.060 | -0.895 | 0.371 | 172.41 |
| Diptera Abundance | NULL | 5.092 | 0.11 | 46.3 | --- | 184.93 |
|  | Humidity + Site Type | 0.027 | 0.015 | 1.833 | 0.032 | 185.38 |
|  |  | -0.390 | 0.182 | -2.142 | 0.032 |  |
|  | Humidity | 0.03 | 0.017 | 1.749 | 0.08 | 185.75 |
|  | Perimeter-Area Ratio | -0.116 | 0.082 | -1.409 | 0.159 | 186.24 |
|  | Perimeter-Area Ratio +  Site Type | -0.011 | 0.104 | -0.110 | 0.912 | 187.61 |
|  |  | -0.399 | 0.264 | -1.514 | 0.130 |  |
|  | Humidity +  Perimeter-Area Ratio | 0.278 | 0.016 | 1.710 | 0.087 | 187.80 |
|  |  | -0.107 | 0.078 | -1.372 | 0.170 |  |
|  | Humidity × Site Type | 0.041 | 0.040 | -1.033 | 0.302 | 188.79 |
|  | Humidity × Perimeter-Area Ratio | 0.026 | 0.023 | 1.149 | 0.251 | 190.88 |
|  | Perimeter-Area Ratio × Site Type | -0.150 | 0.223 | -0.671 | 0.502 | 191.48 |
| Hymenoptera Richness | NULL | 2.404 | 0.075 | 31.98 | --- | 75.01 |
|  | Perimeter-Area Ratio | 0.064 | 0.059 | 1.09 | 0.276 | 76.47 |
|  | Perimeter-Area Ratio + Site Type | 0.103 | 0.081 | 1.266 | 0.205 | 79.06 |
|  |  | -0.145 | 0.208 | -0.698 | 0.485 |  |
|  | Perimeter-Area Ratio × Site Type | -0.055 | 0.172 | -0.318 | 0.750 | 82.59 |
| Hemiptera Richness | NULL | 1.447 | 0.121 | 11.93 | --- | 62.75 |
|  | No. Purple Flowers | -0.011 | 0.009 | -1.184 | 0.236 | 63.93 |
|  | No. Purple Flowers  + Site Type | -0.009 | 0.010 | -0.918 | 0.359 | 64.70 |
|  |  | -0.376 | 0.250 | -1.501 | 0.133 |  |
|  | No. Purple Flowers × Site Type | -0.001 | 0.023 | -0.030 | 0.976 | 68.34 |
| Diptera Richness | NULL | 1.812 | 0.101 | 17.94 | --- | 64.39 |
|  | No. Total Flowers | -0.003 | 0.003 | -1.02 | 0.308 | 65.91 |
|  | No. Total Flowers × Site Type | -0.003 | 0.003 | -0.954 | 0.340 | 68.99 |
|  |  | -0.007 | 0.214 | -0.033 | 0.974 |  |
|  | No. Total Flowers × Site Type | 0.001 | 0.009 | 0.073 | 0.942 | 72.62 |
